# Imaging Characteristics in the Ataxia-Neuropathy Spectrum Related to POLG Mutation

**DOI:** 10.7759/cureus.116575

**Published:** 2026-09-20

**Authors:** Abraham Alhatou, Omar M Alhatou, Mohamed Ghamoodi, Mohammed Alhatou

**Affiliations:** 1 Medical School, Lincoln Memorial University - DeBusk College of Osteopathic Medicine, Knoxville, USA; 2 Medical School, Royal College of Surgeons in Ireland - Bahrain, Bahrain, BHR; 3 Neurology Department, Hamad Medical Corporation, Al-Khor, QAT; 4 Neurology Department, Hamad Medical Corporation, Doha, QAT

**Keywords:** ataxia-neuropathy spectrum, dorsomedial thalamus, mitochondrial disease, polg mutation, sensory ataxia, w748s

## Abstract

Mutations in the mitochondrial DNA polymerase gamma (POLG) gene cause a broad spectrum of neurodegenerative disorders, including the ataxia-neuropathy spectrum (ANS). Substantial phenotypic heterogeneity and overlapping clinical features can delay diagnosis. We report a 38-year-old woman with no significant past medical history who presented with a three-year history of slowly progressive sensory ataxia that worsened in the dark. Examination revealed mild dysarthria, large-fiber sensory loss, and a positive Romberg sign. Extensive metabolic, toxic, autoimmune, and infectious work-up was unrevealing. Nerve conduction studies confirmed a mild peripheral sensory neuropathy. Brain magnetic resonance imaging (MRI) demonstrated distinct bilateral, symmetric T2-weighted and fluid-attenuated inversion recovery (FLAIR) hyperintensities in the dorsomedial thalami and cerebellar white matter. Whole-exome sequencing confirmed a homozygous W748S mutation in the POLG gene, establishing a diagnosis of POLG-related ANS. This imaging pattern, though not specific to POLG-related disease, may support earlier consideration of targeted genetic testing when present alongside a compatible clinical picture.

## Introduction

The POLG gene, located on chromosome 15q26.1, encodes the catalytic subunit of DNA polymerase gamma, the enzyme responsible for replication and repair of mitochondrial DNA [1]. Mutations in this gene give rise to the so-called POLG-related disorders, a continuum that includes Alpers-Huttenlocher syndrome, myoclonic epilepsy myopathy sensory ataxia (MEMSA), chronic progressive external ophthalmoplegia, parkinsonism, male infertility, and the ataxia-neuropathy spectrum (ANS) [1,2]. ANS itself represents a unifying diagnostic category that has superseded two historically distinct designations, mitochondrial recessive ataxia syndrome (MIRAS) and sensory ataxic neuropathy, dysarthria, and ophthalmoparesis (SANDO), which are now recognized as overlapping presentations along the same phenotypic continuum [3].

ANS represents a major adult-onset phenotype, characterized primarily by sensory ataxia, peripheral neuropathy, and variable central nervous system involvement [2]. While generalized cerebral or cerebellar atrophy is a common but nonspecific finding, specific spatial patterns of parenchymal involvement, particularly within the deep gray matter and brainstem nuclei, have been described as diagnostic markers in a small number of previously reported POLG-ANS cases [4]. Timely recognition of POLG-related disorders is essential, as affected individuals are at increased risk of refractory epilepsy and valproate-induced hepatotoxicity, underscoring the practical importance of early genetic testing [3]. Furthermore, establishing the genetic diagnosis is necessary for accurate diagnostic guidance, multidisciplinary anticipatory care, and family planning through reproductive risk stratification and carrier screening. This report illustrates a presentation of adult-onset ANS associated with a homozygous POLG W748S mutation, with an emphasis on its MRI findings.

## Case presentation

A 38-year-old woman with no significant past medical history was referred to the neurology clinic for a three-year history of slowly progressive gait instability. The imbalance was markedly worse at night and with eye closure. She denied muscle weakness, diplopia, other visual disturbances, or seizure activity.

On examination, mental status and cranial nerve testing were normal apart from mild dysarthria; ocular motility was full without ophthalmoplegia or nystagmus, bedside audiometric testing (Weber and Rinne) was intact, and motor strength was fully preserved throughout. Deep tendon reflexes were symmetric but diminished (1+) with trace ankle jerks. Sensory testing revealed significantly diminished joint position and vibration sense to the level of the bilateral ankles. Coordination testing showed a mild bilateral intention tremor, and the Romberg sign was positive. The autonomic nervous system was intact. There was no family history of consanguinity.

Initial laboratory work-up aimed at excluding toxic, metabolic, and autoimmune etiologies was unremarkable, including liver function panels, serum vitamin B12, vitamin E, thyroid hormones, antinuclear antibodies, paraneoplastic antibodies, and vasculitis screens. Infectious serologies were also negative.

Nerve conduction studies demonstrated a mild peripheral sensory neuropathy, with symmetrically reduced sensory nerve action potential amplitudes. Needle electromyography of the lower extremities was normal, arguing against an active myopathic or neurogenic motor process.

Brain MRI demonstrated distinct, bilateral, and symmetric hyperintensities on T2-weighted and FLAIR sequences localized to the dorsomedial thalamic nuclei (Figure 1A) and the deep cerebellar white matter (Figure 1B). There was no evidence of inferior olivary nucleus involvement or hypertrophy.

**Figure 1:**
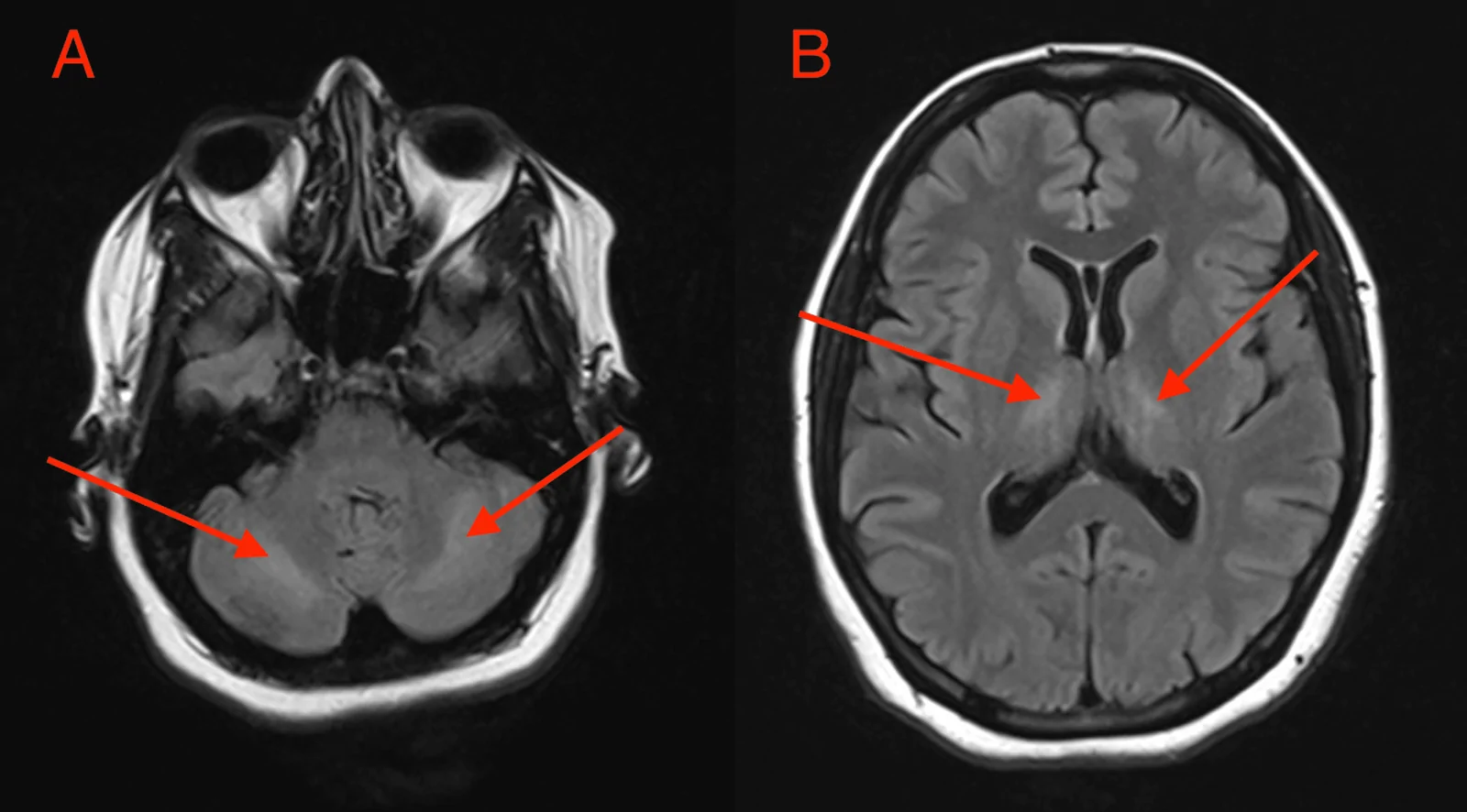
Axial FLAIR/T2 brain MRI Axial FLAIR/T2 brain MRI showing characteristic bilateral, symmetric hyperintensities (red arrows) within the dorsomedial thalami (A) and deep cerebellar white matter (B), without inferior olivary nucleus involvement.

Given the combination of sensory neuropathy, progressive ataxia, and this specific pattern of deep gray matter signal change, a primary mitochondrial etiology was strongly suspected. Whole-exome sequencing (GeneDx; mean coverage depth 116x, 98.7% of target bases covered at a minimum of 10x) confirmed a homozygous pathogenic variant in the POLG gene (Table 1): c.2243G>C (p.Trp748Ser) in exon 13 (NM_002693.2), corresponding to the W748S substitution, at chromosomal position 15:89866657, catalogued in the Single Nucleotide Polymorphism Database (dbSNP) as rs113994097. The variant was confirmed by an orthogonal sequencing method. Testing for mitochondrial DNA point mutations and deletions was negative, supporting a nuclear-encoded rather than primary mitochondrial DNA etiology. Segregation testing showed that the patient's two unaffected sons were both heterozygous carriers of the variant, while her partner did not carry it, consistent with autosomal recessive inheritance in this family. Together, these findings established a definitive diagnosis of POLG-related ANS.

**Table 1:** Genetic testing findings

Parameter	Finding
Gene	POLG
Variant (cDNA)	c.2243G>C
Variant (protein)	p.Trp748Ser (W748S)
Exon / transcript	Exon 13 / NM_002693.2
Chromosomal position (GRCh37/hg19)	15:89866657
dbSNP ID	rs113994097
Zygosity	Homozygous
Classification (GeneDx clinical laboratory)	Pathogenic variant
Population frequency (Genome Aggregation Database, gnomAD)	0.10% (285/277,084 alleles); no homozygotes reported
In silico prediction (protein variation effect analyzer)	-5.25 (deleterious)
Sequencing depth / confirmation	116x mean coverage; confirmed by orthogonal method

## Discussion

The W748S substitution is a well-characterized European founder mutation and one of the most frequent pathogenic POLG variants underlying inherited ataxia in populations of European descent [2,5,6]. As in this patient, ANS typically manifests in early-to-middle adulthood with sensory ataxia from large-fiber sensory neuropathy, together with progressive cerebellar dysfunction [2].

While laboratory testing and electrophysiology help characterize the peripheral neuropathy, neuroimaging remains central to localizing central nervous system involvement. Early in the disease course, brain MRI is frequently normal or shows only nonspecific mild cerebellar atrophy. As the disease progresses, however, lesions have been described at metabolically vulnerable sites in a limited number of reported cases: bilateral, symmetric T2/FLAIR hyperintensities in the dorsomedial thalami and deep cerebellar white matter are thought to reflect localized mitochondrial energy failure, edema, and subsequent gliosis [4,7]. Similar thalamic and cerebellar signal changes are not exclusive to POLG-related disease and have been described in other metabolic and neurodegenerative conditions [8], so this pattern should be interpreted alongside the broader clinical picture rather than in isolation.

Another well-described neuroradiological feature of POLG disease is structural change of the inferior olivary nuclei, manifesting as hypertrophic olivary degeneration (HOD) with T2 hyperintensity [4]. Although HOD was absent in our patient, the combination of dorsomedial thalamic lesions and symmetric cerebellar white matter change, in the context of a progressive degenerative ataxia, is consistent with an underlying POLG mutation and may help prioritize confirmatory genetic testing alongside standard evaluation for alternative causes of ataxia.

The W748S variant identified in this patient is notably rare, observed in only 0.10% of alleles across the Genome Aggregation Database (gnomAD), which incorporates and extends the earlier Exome Aggregation Consortium (ExAC) cohort reported by Lek et al., with no homozygous individuals reported in either dataset [9,10]. Variant classification in this case followed the assessment of the clinical testing laboratory (GeneDx) rather than ClinVar, which lists conflicting interpretations for this variant; supporting evidence included its rarity in reference population databases and a deleterious in silico prediction by protein variation effect analysis. Such discrepancies between clinical laboratory classification and public variant databases are not uncommon for POLG variants and underscore the importance of interpreting genetic results alongside detailed clinical and radiographic correlation, as was performed in this case, rather than relying on molecular findings in isolation. Segregation analysis in this family, showing heterozygous unaffected carrier sons and a non-carrier partner, further supported an autosomal recessive pattern and strengthened confidence in the causal role of this variant.

## Conclusions

In patients presenting with progressive, otherwise unexplained ataxia associated with sensory neuropathy, structural neuroimaging can help refine the diagnostic pathway. This imaging pattern may raise suspicion for POLG-related disease in the appropriate clinical context, though it is not specific to this diagnosis and its predictive value remains to be established in larger cohorts. Familiarity with this radiographic pattern should prompt consideration of targeted genetic testing for POLG mutations when clinically appropriate.
